# A Multi-Level Interdependent Hierarchy of Interpersonal Synergies in Team Sports: Theoretical Considerations

**DOI:** 10.3389/fpsyg.2021.746372

**Published:** 2021-12-03

**Authors:** Rodrigo Santos, Pedro Passos

**Affiliations:** ^1^Departamento de Educação Física, Universidade Federal de Viçosa, Viçosa, Brazil; ^2^Centro Interdisciplinar de Estudo da Performance Humana (CIPER), Faculdade de Motricidade Humana, Universidade de Lisboa, Lisbon, Portugal

**Keywords:** interpersonal coupling, task-specificity, coordination, degeneracy, team sports

## Introduction

Most daily activities (e.g., dialoguing, road trafficking) entails constant coordination between humans. Interpersonal coordination may occur when people share similar goals (Schmidt and Richardson, 2008; Davis et al., 2016) and results from the haptic or visual perceptive coupling of relatively independent individuals (e.g., a figure skating duo, two competing tennis players), which temporarily form a single coherent unit, defined as a soft-assembled *interpersonal synergy* (Black et al., 2007; Riley et al., 2011). Synergies are the basic structures from which movement coordination emerges, as they provide individuals with proper means for dealing with complex behavior under countless circumstances (Kelso, 2021).

As in other movement systems, the identification of soft-assembled synergies in team sports is often grounded on two key properties: dimensional compression and reciprocal compensation (Riley et al., 2011). Dimensional compression refers to the process through which the coupling of the previously independent degrees of freedom (DoF) (e.g., players) within a system are reduced to a smaller number, resulting in a low-dimensional functional unit (Bingham, 1988; Riley et al., 2011). For instance, instead of describing the behavior of each individual element, it describes the behavior of a dyad composed by two coupled elements, which consequently decreases the DoF under analysis. In turn, reciprocal compensation describes the capacity of each player within a synergy to adjust their behavior (e.g., increase or decrease running velocity), to compensate the behavioral variability among teammates, in order to stabilize a performance variable (e.g., interpersonal distance) (Riley et al., 2011; Araújo and Davids, 2016).

In team sports, conceptualized as complex adaptive systems, it has already been suggested that interpersonal synergies are formed through feedback loops from the environment to the players and vice versa (Balagué et al., 2019). This suggests the existence of nested synergies, where some synergies are formed “within” others. A recent study investigating a cooperative dyadic slackline task displayed evidence of a nested organization between intrapersonal and interpersonal synergies, with the intrapersonal synergies at a lower level (i.e., formed within other higher-level synergies) of organization (Montull et al., 2021). Hence, attempts to identify interpersonal coordination patterns that account for performance outcomes should consider the specificities of (nested) synergy formation at the following levels of analysis: micro, dyadic level (e.g., among two teammates); meso, group level (e.g., intra-group) and; macro, collective level (e.g., intra-team) carried out through circular causality (Juarrero, 1999; Davids, 2015). Circular causality in dynamical systems was defined as a situation in which the cooperation among individual elements of a system influence the global system behavior, which, in turn, governs the behavior of these individual elements (Kelso, 1995). While the nested synergies concept implies a more fixed hierarchy, the idea of circular causality (not to be confused with hierarchy) suggests a more dynamic behavior of that hierarchy. For instance, in the course of a football match, sometimes synergies may be formed between two defenders (dyadic level) and influence the soft-assembling of synergies of an entire defensive sector, whereas at other times this meso-level of the defensive sector may influence how synergies between two defenders will be formed. That is why we suggest a “multi-level interdependent hierarchy” in the soft-assembling of interpersonal synergies.

The interdependence among these functional levels entails that only dimensional compression and reciprocal compensation may not suffice to accurately account for the principles that govern the formation of interpersonal synergies in team sports. Literature has recently proposed that degeneracy, defined as “the ability of elements that are structurally different to perform the same function or yield the same output” (Edelman and Gally 2001, p. 98), is likely a primary feature of soft-assembled synergies (Glazier and Davids, 2009; Araújo and Davids, 2016; Pol et al., 2020). In sports, these elements can be described as individual players, dyads or even groups of players. Therefore, there are several elements which are able to perform the same task/function, for instance if a full back fails to prevent an opposing winger from progressing toward the penalty area, another player (e.g., a center back) may be capable of fulfilling his/her role. In this paper, we suggest that these features allow to infer that coordination dynamics in team sports display task-specific hierarchies, which means that at every instant any of the three levels can “lead” the coordination dynamics within a team sports match.

Despite the growing interest on this topic, only recently has research raised awareness of the formation of interpersonal synergies in sports as a task-specific phenomenon with some kind of hierarchy (Balagué et al., 2019; Pol et al., 2020; Montull et al., 2021). This means that some important theoretical and methodological gaps regarding the hierarchical organization of interpersonal synergies in team sports still need to be bridged. Therefore, the purpose of this opinion article is to provide some theoretical considerations that support interpersonal synergies in team sports as task-specific phenomena with a multi-level interdependent hierarchical organization.

## A Multi-Level Interdependent Hierarchy of Synergies in Team Sports

In team sports, the ultimate purposes of a team during the course of match are to score points (or goals) and to prevent the opposition from scoring. These purposes induce teams to create scoring opportunities through coordinated movements, and to preserve defensive balance and organization (Gréhaigne et al., 1999). Consequently, acting in a coordinated and functional fashion enables players to deal with the continuously emerging goals (e.g., preventing an opponent from approaching a potentially risky area) that lead to scoring/avoiding points or goals (Araújo et al., 2015).

The dynamics of team sports performance imply that whenever an interpersonal synergy is unable to deal with contextual demands a “non-functional” synergy is assembled. Synergies are formed whenever coordination arises, although certain synergies may not fit the contextual demands, which will likely lead to performance decrements and/or unsuccessful outcomes. Therefore, players need to adapt to form new (and more functional) synergies, either to fulfill the ongoing task or in response to newly formed environmental constraints (Latash, 2008; Kelso, 2021). Accordingly, a specific performance goal (e.g., reducing the space, to decrease the opportunities of action of an opponent in possession of the ball) can be attained by the assembly of interpersonal synergies at distinct levels, such as dyadic or group levels (e.g., four players within a defensive set) (Balagué et al., 2019). By enabling different players to perform the same role (e.g., maintain an interpersonal distance to a teammate), degeneracy is an indispensable property to be accounted for in the analysis of synergistic behavior in team sports, since it allows reciprocal compensation and, consequently, dimensional compression (Araújo and Davids, 2016; Hristovski and Balagué, 2020). However, due to task constraints, players can switch roles from one moment to the next. For instance, in one moment a defender needs to stabilize the interpersonal distance to a teammate, in a dyadic behavior, with the purpose of preventing the player in possession to progress toward the goal, whereas in the next timestep she/he needs to stabilize the interpersonal distance to the closest teammates, so as to preserve a relative stability of the defensive line, in a group behavior with purposes that also include restraining the opponents' progression toward the goal. Interpersonal couplings at different levels interact with and overlap each other (e.g., the same player can contribute simultaneously to a dyadic and to a group synergy) to achieve specific performance goals (Montull et al., 2021). Therefore, since all the players within a team should be able to perform similar tasks (e.g., maintain a suitable interpersonal distance), degeneracy plays an essential role in the interdependence among levels of hierarchical organization of synergies. Nevertheless, the question regarding which synergy level “leads” the match dynamics remains unanswered.

Addressing such a complex issue requires summoning up the inherent principles of hierarchical synergic control, whether in multi-agent systems, such as team sports, or in less complex ones, such as human hands and fingers (Latash and Zatsiorsky, 2016). For example, after analyzing intrapersonal synergistic behavior in prehensile tasks, Gorniak et al. (2007a,b) identified the presence of idiosyncratic behaviors among synergies at two distinct hierarchical levels (i.e., hands level and fingers level) for producing force. Specifically, the authors observed that during prehensile tasks using only one hand, stabilization of the total force was achieved through the formation of strong synergies at the fingers level. Conversely, in prehensile tasks in which participants were asked to use their two hands, comparably strong synergies were formed between both hands, yet not between the fingers (Gorniak et al., 2007a,b). This suggests that the hands level (i.e., meso level) overlapped the fingers level (i.e., micro level) regarding synergy formation and strength at an intrapersonal scale. Thus, by hierarchical control we mean the existence (even if temporary) of a higher level, which constrains the behavior of elements at lower levels.

If interpersonal synergies conform to the same governing rules as those at the intrapersonal level (Riley et al., 2011), the aforementioned findings could precipitate the idea that soft-assembled interpersonal synergies involving more players result in the weakening of lower level synergies. For instance, a synergy formed between two center-backs “disappears” due to a new intra-sectorial synergy, formed by the whole defensive set. Nevertheless, a more likely explanation for the example above is that the lower level synergy formed by the two center-backs does not “disappear” but to keep the functionality, turns its focus to the stabilization of another performance variable, for instance from the interpersonal distance to the interpersonal angle, probably as a result of changes in task constraints (Fajen, 2005; Balagué et al., 2019). In fact, changing task characteristics may lead to a reorganization of the hierarchical levels of a synergy, so that the levels with higher functionality to achieve a specific performance goal are prioritized. For instance, two players forming a dyadic (micro-level) synergy aiming to regain ball possession can subsequently give up on their initial task goal, yet preserve the synergy while directing the focus to how to reestablish the balance of the whole defensive sector – a meso-level synergy (Bingham, 1988).

New task definitions emerge as a result of match dynamics, and consequently some performance variables can become more relevant to synergy formation. Performance variables characterize the performance goals that players seek to stabilize together (e.g., interpersonal distances or angles). Thus, it seems appropriate to suggest that the hierarchical organization of synergies is more closely related to these “new” performance variables, than to the number of players involved *per se*. If so, it will be up to the task characteristics to determine the hierarchical organization of synergies. Therefore, we hypothesized that the formation of functional (i.e., successful) interpersonal synergies in team sports is governed by a task-specific, multi-level interdependent hierarchy. To deal with this sort of hierarchy, degeneracy plays a relevant role. A system in which all the structurally different elements (i.e., players) are able to perform the same role (e.g., stabilize interpersonal distances or angles) are more likely to have the necessary conditions to adapt and achieve the same outcome by assembling synergies to successfully perform tasks at all levels.

Preliminary findings in soccer (association football), reported by Carrilho et al. (2020), revealed that synergies formed by different numbers of players generated distinct values of the performance variable (team synchronization). The authors investigated two opposing teams during a match, and compared synchronization values between different team configurations (i.e., groups of players), assuming these configurations as different synergistic levels. Although only initial humble steps have been taken in this direction, the current empirical evidence has thus far reinforced the essential role of degeneracy in addressing a multi-level interdependent hierarchy of interpersonal synergies in team sports.

Thus, we suggest that addressing the issue of a multi-level interdependent hierarchy of interpersonal synergies requires identifying the most relevant performance goals for the three levels of analysis (i.e., micro, meso, and macro) and for every phase of play in a team sports match. Some examples of relevant performance variables include players' interpersonal distance within a dyad (the micro level), the area of a polygon created by the players within a group (the meso level) and the distance between a team's centroid and the goal (the macro level). These variables can be measured using players' positional data allowing to capture interpersonal synergies for the three levels of analysis (please see Passos et al., 2018 for further detail). As for how to capture the interdependencies among synergy levels, despite being a tad early to define a method for this purpose, we suggest that addressing this issue by using a cluster analysis might be a promising start, as Montull et al. (2021) have already ascertained in their study with an interpersonal slackline task. However, the key issue is: How to measure the relevance of a performance variable? This issue remains unresolved!

## Discussion

The aim of this opinion article was to evoke key assumptions supporting the hypothesis of interpersonal synergies as a multi-level, interdependent, hierarchically organized phenomena, as well as to discuss their implications for studying multi-level synergistic behavior in team sports. Here we may suggest that task-specificity constrains the formation of synergies at all levels. Accordingly, identifying the hierarchical organization of interpersonal synergies in team sports entails the adoption of a perspective of synergy formation as a task-dependent phenomenon. However, what needs to be discussed is, how do task characteristics prioritize certain levels over others? Do stronger synergies constrain (overlap) weaker synergies, or are the levels defined by the number of elements that assemble a synergy? All these questions remain unanswered. Previous research in football (Carrilho et al., 2020) provided us with some thought-provoking queries, regarding the role played by performance goals in synergy formation and strength at each level, as mentioned throughout this paper. Therefore, we suggest that future attempts to address these questions should be equated with the relevance of the performance goals.

Finally, investigating the hierarchical organization of synergies in team sports, as well as the relationship among the distinct synergistic levels may help researchers and coaches in understanding, planning, practicing and assessing team performance, as well as the effectiveness of styles of play.

## Author Contributions

RS and PP: article development and composition. PP: final draft review. All authors contributed to the article and approved the submitted version.

## Funding

This study was financed in part by the Coordenação de Aperfeiçoamento de Pessoal de Nível Superior—Brasil (CAPES)—Finance Code 001.

## Conflict of Interest

The authors declare that the research was conducted in the absence of any commercial or financial relationships that could be construed as a potential conflict of interest.

## Publisher's Note

All claims expressed in this article are solely those of the authors and do not necessarily represent those of their affiliated organizations, or those of the publisher, the editors and the reviewers. Any product that may be evaluated in this article, or claim that may be made by its manufacturer, is not guaranteed or endorsed by the publisher.
